# Effect of annealing treatments on photoluminescence and charge storage mechanism in silicon-rich SiN_*x*_:H films

**DOI:** 10.1186/1556-276X-6-178

**Published:** 2011-02-28

**Authors:** Bhabani Shankar Sahu, Florian Delachat, Abdelilah Slaoui, Marzia Carrada, Gerald Ferblantier, Dominique Muller

**Affiliations:** 1 InESS-UdS-CNRS, 23 Rue du Loess, 67037 Strasbourg, France

## Abstract

In this study, a wide range of a-SiN_***x***_:H films with an excess of silicon (20 to 50%) were prepared with an electron-cyclotron resonance plasma-enhanced chemical vapor deposition system under the flows of NH_3 _and SiH_4_. The silicon-rich a-SiN_*x*_:H films (SRSN) were sandwiched between a bottom thermal SiO_2 _and a top Si_3_N_4 _layer, and subsequently annealed within the temperature range of 500-1100°C in N_2 _to study the effect of annealing temperature on light-emitting and charge storage properties. A strong visible photoluminescence (PL) at room temperature has been observed for the as-deposited SRSN films as well as for films annealed up to 1100°C. The possible origins of the PL are briefly discussed. The authors have succeeded in the formation of amorphous Si quantum dots with an average size of about 3 to 3.6 nm by varying excess amount of Si and annealing temperature. Electrical properties have been investigated on Al/Si_3_N_4_/SRSN/SiO_2_/Si structures by capacitance-voltage and conductance-voltage analysis techniques. A significant memory window of 4.45 V was obtained at a low operating voltage of ± 8 V for the sample containing 25% excess silicon and annealed at 1000°C, indicating its utility in low-power memory devices.

## Introduction

Silicon nitride-based dielectrics are drawing considerable attention because of their utility in a wide variety of electronic and optoelectronic applications due to their compatibility with the existing mainstream CMOS technology and tunable emission in visible range, which can be applied for developing non-volatile memories and Si-based light-emitting diodes [1-6]. The field strength is needed for electroluminescence in SiO_*x *_close to the breakdown strength of silicon oxide (6-10 MV/cm) [7,8]. In this regard, SiN_*x *_can be a better choice, as the field needed to inject electrons and holes (2-4 MV/cm) is weaker than that of SiO_2_, and is much lower than the breakdown strength of Si_3_N_4 _(9 MV/cm) [9,10]. Even if the band gap of Si_3_N_4 _is approximately 5.3 eV (8.2 eV for SiO_2_), it should be sufficient to confine charge carriers into SiN_*x *_matrix [11]. In addition, SRSN also contains a high density of deep level electron and hole traps, which can give rise to a strong trapping of electrons and holes, indicating its utility for light-emitting as well as charge storage devices. Another major interest for using silicon nitride rather than silicon oxide is the possibility of obtaining efficient emission at relatively shorter wavelength. Several authors have reported intense luminescence in blue-green region, which is very rare in silicon oxide-based materials [12,13] and therefore provides the possibility for fabricating full-color devices based on silicon technology. Previous studies revealed that the PL from silicon nitride films can be significantly enhanced with the introduction of a thermal SiO_2 _buffer interlayer and subsequent high-temperature annealing in N_2 _ambient [14]. However, it is still unclear at which annealing temperatures the PL is the strongest, and how does this annealing temperature depend on excess of Si in the silicon-rich silicon nitride (SRSN) films, and what physical mechanisms are responsible for the PL enhancement.

In addition, SiN/SiO_2 _stack structures have wide applications in non-volatile charge memories (NVMs). Their use started since early 1970s as metal-nitride-oxide-silicon (MNOS) structures [15], and their various derivatives, such as metal-oxide-nitride-oxide-silicon (MONOS), silicon-nitride-oxide-silicon (SNOS), silicon-oxide-nitride-oxide-silicon (SONOS), metal-nitride-nitride-silicon (MNNOS) structures have remained as the state-of-art techniques for NVMs [16]. SiN_*x*_-based memories are inexpensive, highly integrated, and can be expanded to store two bits of data per memory cell [17]. Recently, Mine et al. [18] have proposed the use of SRSN layer as the charge-trapping layer instead of stoichiometric Si_3_N_4 _layer with enhanced memory properties. With further scaling down of the device dimensions, the SiN_*x*_/SiO_2 _interface plays the vital role for the large amount of captured charges, and these can be attributed to the excess of silicon at these interfaces, which can capture both electrons and holes. Furthermore, with suitable choice of excess silicon and annealing treatment, the formation and evolution of silicon nanoparticles (Si-nps) in the SRSN charge-trapping layer can be possible. The presence of well-separated Si-nps can act as discrete charge storage nodes, thereby improving the leakage current and retention time. The fabrication of uniform, reproducible, and tunable Si nanostructures by simple and flexible technique, compatible with existing CMOS technology, is extremely important. In this regard, one of the best methods used for the evolution of Si-ncs in silicon nitride/oxide-based dielectrics is the high-temperature annealing of these films containing excess amount of Si. One of the major issues is the inevitable interface states at the crystalline silicon/dielectric interface. For the improvement of the storage properties, these interface states should be minimized. In addition, the size, density, and separation between the Si-nps should be optimized.

In this study, SRSN layers with different excess amount of Si were utilized for the fabrication of Si_3_N_4_/SRN/SiO_2_/Si (NNOS) structures. The effect of Si-excess and post-deposition annealing temperature on photoluminescence (PL) and charge storage mechanism in these stack layers were studied in detail.

## Experimental details

Silicon oxide/silicon-rich silicon nitride/stoichiometric silicon nitride (SiO_2_-SRSN-Si_3_N_4_) stacks were made on p-Si (100) substrates. Before SRSN deposition, 10 nm of thermal oxide was grown on p-Si substrate. Subsequently, SRSN layers of 50 nm were deposited by electron-cyclotron resonance plasma-enhanced chemical vapor deposition (ECR-PECVD) under the flows of silane (SiH_4_) and ammonia (NH_3_) at 300°C under a microwave (MW) power of 300 W. In this experiment, the flow of SiH_4 _was fixed at 14 sccm, while the chemical compositions of the films were controlled by varying the NH_3 _flow. The top stoichiometric silicon nitride films were deposited with the same ECR-PECVD system under a MW power of 500 W and an RF power of 8 W at 300°C. Rutherford backscattering (RBS) and elastic recoil detection analysis (ERDA) techniques were used to determine the Si/N ratio and hydrogen content in the films, respectively. Fourier transform infrared (FTIR) spectra were recorded using Bruker-EQUINOX55 spectrophometer. PL spectra were recorded at room temperature using a 355-nm exciton wavelength of a Nd:YVO_4 _laser. The emitted light was detected by a BWTek BCR 112E spectrometer coupled with a Sony ILX511 CCD linear image sensor. The response of the detection system was precisely calibrated with a tungsten wire calibration source. Energy-filtered transmission electron microscopy (EFTEM) and high-resolution transmission electron microscopy (HREM) observations were performed using a JEOL2100F microscope operating at 200 kV equipped with a corrector for spherical aberration and the last generation of Gatan-imaging filter. The EFTEM is formed by the electrons which are selected by a slit placed in the energy dispersive plane of the spectrometer at 17 eV with a width of ± 2 eV. Al-Si_3_N_4_-SRSN-SiO_2_-Si (MNNOS) memory capacitor structures were fabricated from the samples by evaporating Al electrodes of 0.8-mm diameter with a shadow mask and Al rear-side contact after scratching the back surface. Capacitance-voltage (*C*-*V*) and conductance-voltage (*G*-*V*) measurements were carried out using HP4192A impedance analyzer through a LABVIEW interface.

## Results and discussion

### Composition of MW-CVD SRSN films

**Figure 1:**
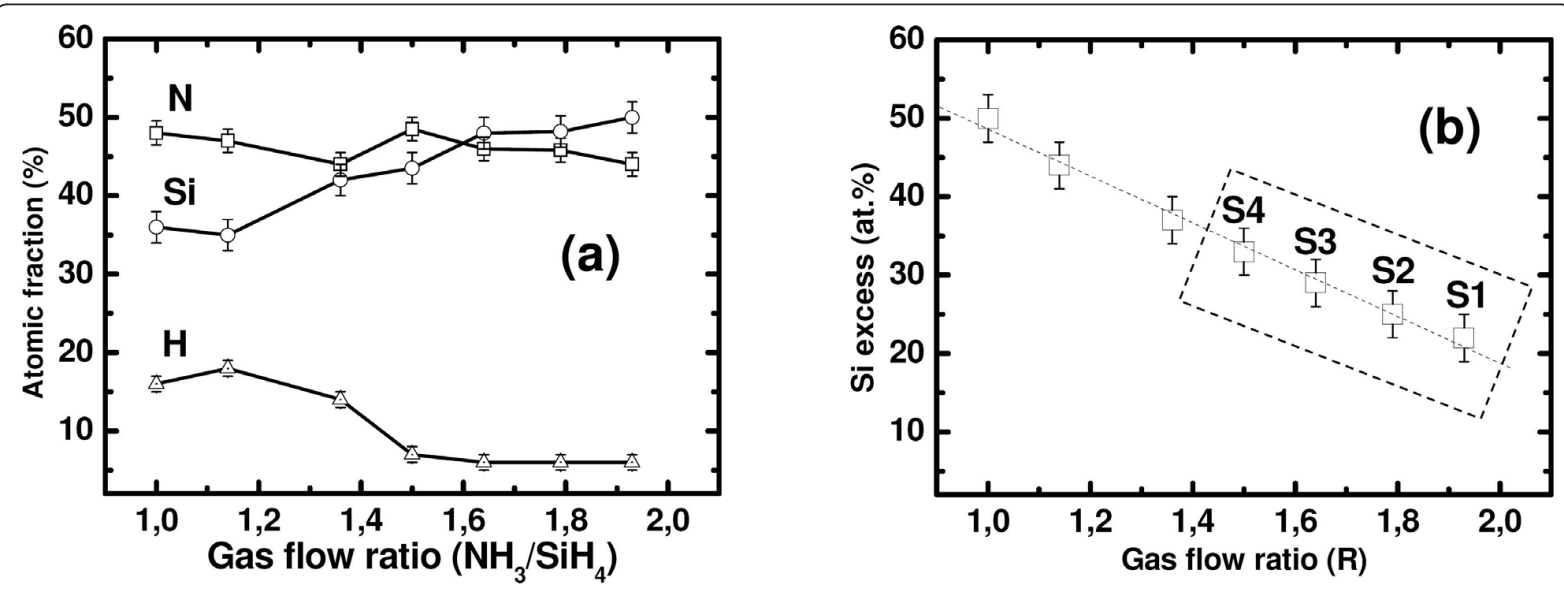
**Atomic fraction of Si, N, and H versus gas flow ratio in SRSN films deposited on Si substrates, as measured by RBS and ERDA techniques**.

Figure 1 depicts the variation of atomic content of silicon, nitrogen, and hydrogen, as estimated from RBS and ERDA measurements on 50 nm of SRSN layers deposited on Si-substrates with different gas flow ratios. In particular, the SiN_*x *_samples deposited at a gas flow ratio (R = NH_3_/SiH_4_) of 2.07 (or higher) are close to stoichiometric, whereas samples deposited using lower *R *values are silicon rich, being characterized by excess of Si ranging from 50 to 20 at.% in this study. As evidenced from ERDA measurements, the total amount of hydrogen content decreases from 16 to 6 at.% upon increasing *R *from 1 to 1.93. In the present investigation, the authors have focused on four sets of samples having an excess of Si in the range 33-22 at.%. The samples are defined with notations S1, S2, S3, and S4, where higher numbers refer to higher Si excess in the SiN_*x *_matrix (see Table 1). The samples were further sandwiched between a bottom thermal oxide and a top stoichiometric Si_3_N_4 _for further study.

**Table 1:** Selected samples for investigation

Sample notation	Gas flow ratio (*R *= NH_3_/SiH_4_)	Si atomic excess
S4	1.5	33
S3	1.64	29
S2	1.79	25
S1	1.93	22

### TEM observations of SRSN films

**Figure 2:**
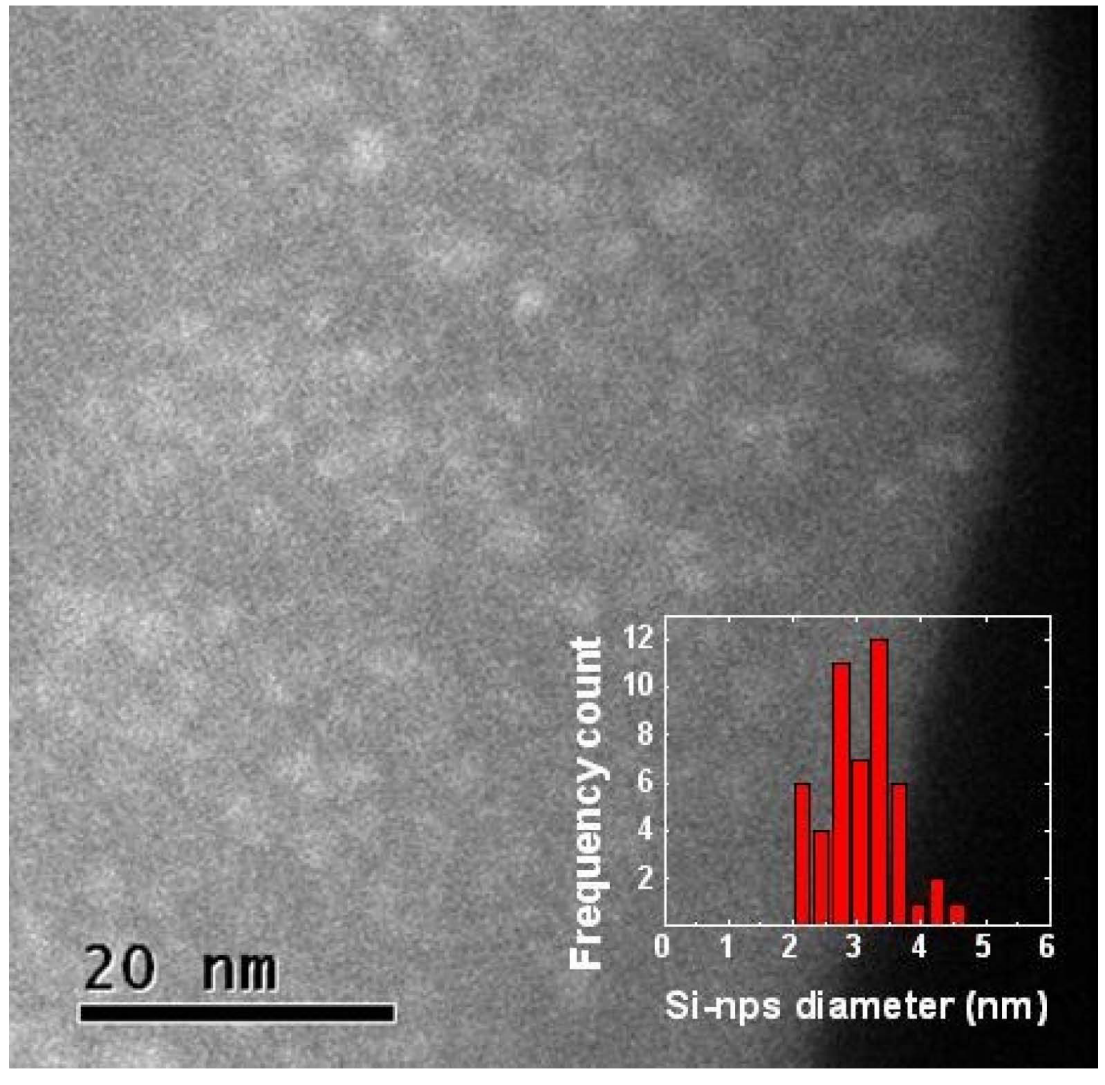
**Cross-sectional EFTEM image of the sample S4 containing 33 at.% of Si excess after annealing at 1100°C for 30 min in N_2 _ambient**.

Figure 2 shows the EFTEM image of 50-nm thick SRSN layer (sample S4) with 33 at.% of Si excess after annealing at 1100°C. High densities of nearly spherical Si-nps are clearly observed. No lattice fringes have been detected in the HREM analysis, suggesting amorphous nature of these nanoclusters. The inset to Figure 2 indicates that the size distribution of Si-nps is centered at 3-nm diameter with a standard deviation of 0.6 nm. However, for other samples with less Si excess, no Si-np was detected in the EFTEM/HREM analysis. It is speculated that even if silicon precipitation has occurred during annealing, the Si cluster size might be below the detection limit of EFTEM (1.5 nm).

### Infrared spectroscopy

**Figure 3:**
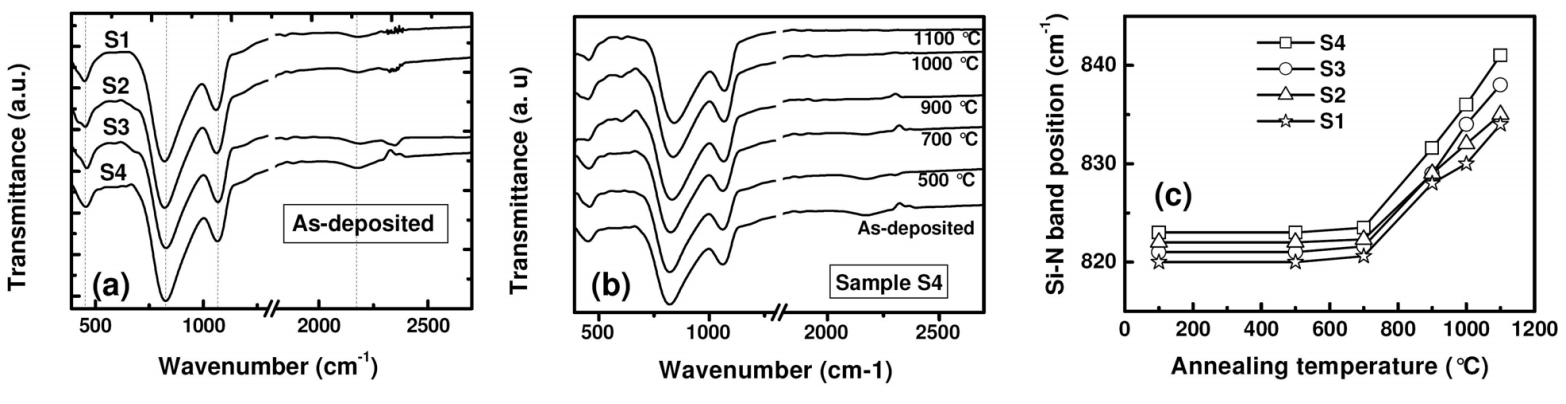
**FTIR transmission spectra **of **(a) **as-deposited Si_3_N_4_/SRSN/SiO_2 _films, **(b) **sample S4 containing 33 at.% of silicon excess and annealed within the temperature range of 500-1100°C for 30 min in N_2 _ambient, **(c) **evolution of Si-N stretching band position of samples S1, S2, S3, and S4 as a function of annealing temperature.

FTIR spectra of as-deposited Si_3_N_4_/SRSN/SiO_2 _structures are shown in Figure 3a. These spectra exhibit the characteristic features of Si-N asymmetric stretching (820 cm^-1^), Si-O-Si stretching (1060 cm^-1^), and Si-H stretching modes (2170 cm^-1^) [19,20]. The peak present around 450 cm^-1 ^can be ascribed to an overlapping of Si-O-Si rocking mode and Si-N breathing mode [21]. No clear feature of N-H stretching mode around 3350 cm^-1 ^is observed. It is speculated that the total amount of N-H bonds (if any) is below the detection limit of FTIR. Previously, Xu et al. [22] have reported that at higher MW power, N-H bonds are less possible to form, survive, and remain in the silicon nitride as they are most likely to be dissociated at a higher MW power [22]. However, the MW power in this study is lower than that used in [22]. Furthermore, Martinez et al. have explained this behavior based on the following chemical reaction [21]:

Si-Si+N-H=Si-H+Si-N

This is a well-known network bond process favored by the tendency to chemical order, whereby formation of Si-H and Si-N bonds is favored at the expenses of Si-Si and N-H bonds. The process is exothermic with a favorable energy balance of 0.25 eV [23]. Also, it can be observed that the intensity of Si-N and Si-H stretching mode increases with increasing Si content of the film following the above reaction kinetics. With increasing Si content, the Si-N peak shifts toward higher wavenumber, indicating enrichment of nitrogen atoms in silicon nitride phase.

Figure 3b shows the FTIR spectra of sample S4 containing 33 at.% of excess silicon and annealed up to 1100°C. FTIR spectra of other samples follow similar trends after the post-deposition annealing treatment. It can be noticed that Si-H bands progressively decreases up to an annealing temperature of 700°C, indicating desorption of hydrogen from the films. At an annealing temperature of 900°C, the Si-H band completely disappears. It can be noticed from Figure 3c that the position of Si-N remains unchanged up to an annealing temperature of 700°C. For higher annealing temperatures, the Si-N peak shifts significantly toward higher wavenumbers. This shift indicates that the number of nitrogen atoms bonded to Si is increasing. Since high temperature annealing process broke the Si-H and N-H bonds and hydrogen effused from the film, the remaining unbonded Si and N atoms could bond together to form new Si-N bonds. Therefore, with increasing annealing temperature, there is enrichment of nitrogen atoms in the silicon nitride phase approximating it to stoichiometric Si_3_N_4_. In addition, temperature-induced evolution of sub-stoichiometric phase present in the as-deposited SRN films toward a biphasic Si/Si_3_N_4 _mixture cannot be ruled out. The phase separation process has been reported for silicon-rich SiO_*x *_films, which also indicates a shift of Si-O band toward higher wave numbers [24,25]. As discussed earlier, a phase separation occurs when SRSN samples are annealed at high temperature (≥ 950°C), resulting in the formation of Si-nps embedded in a nearly stoichiometric silicon nitride matrix [26].

### Photoluminescence

**Figure 4:**
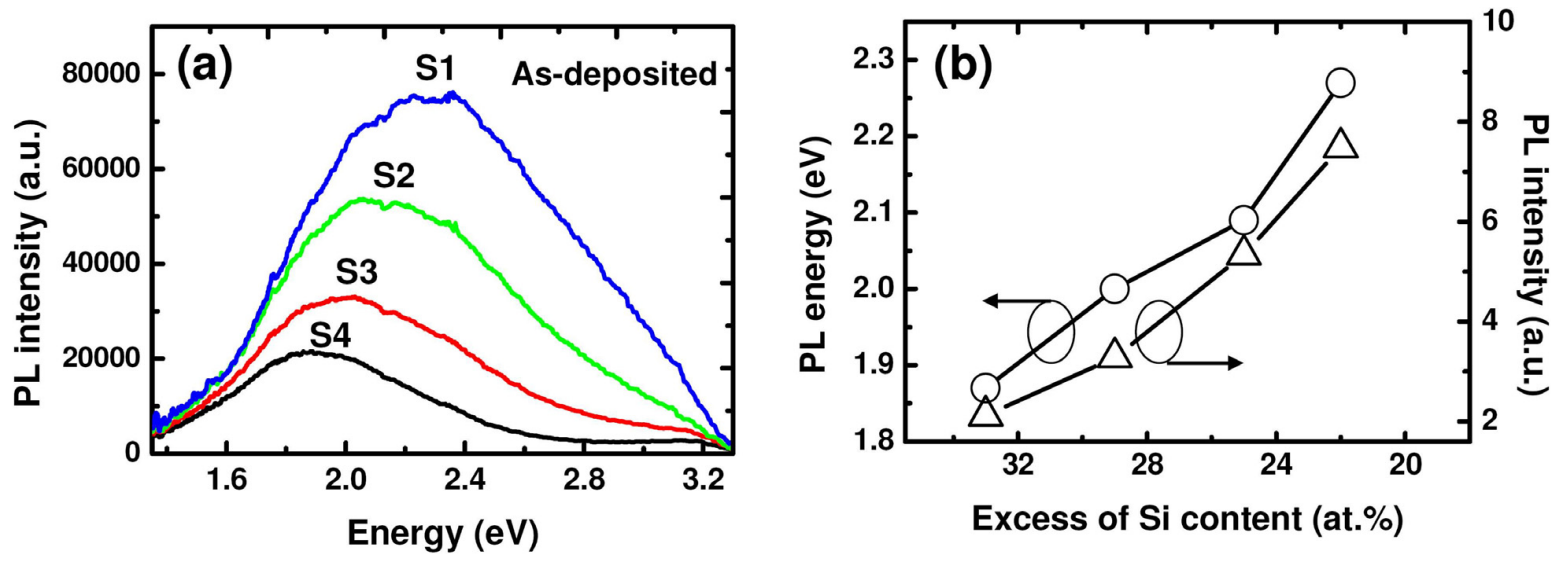
**Roomtemperature PL spectra of as-deposited Si_3_N_4_/SRSN/SiO_2 _films**. **(a) **(Color online) Room temperature PL spectra of as-deposited Si_3_N_4_/SRSN/SiO_2 _films having an excess of silicon from 22 to 33 at.% (samples S1, S2, S3, and S4) in the middle SRSN layer, **(b) **PL energies and intensities of the as-deposited films as a function of silicon excess in the SRSN layer

Figure 4a shows the PL spectra of the as-deposited samples. All the samples exhibit broad and strong visible PL at room temperature. The mechanism for strong luminescence from SiN_*x *_materials is commonly suggested from the combination of Si/SiN_*x *_interface luminescence, gap state luminescence/band-tail luminescence, and luminescence from Si nanodots/clusters. In this investigation, efforts have been made to minimize Si/SiN_*x *_interface luminescence with the introduction of high-quality thermal SiO_2 _before SiN_*x *_deposition, which gives rise to enhanced interface quality with minimal interfacial defect states. Details regarding interface traps will be discussed in the *C*-*V *analysis part. As evident from Figure 4a,b, with increase in nitrogen content in the SRSN films, the PL peak shifts to higher energies together with an increase in the relative intensity and width. This behavior is commonly attributed to a model based on quantum confinement effect (QCE) [27-29]. From the QCE model point of view, PL blue shift is caused by the reduction in Si-np size, and the increase in emission efficiency can be correlated with the onset of pseudo-direct bandgap behavior. However, no sin-np/cluster has been detected in our EFTEM analysis for these as-deposited SRN films. Thus, the role of QCE can be ruled out. The behavior of PL is in very good agreement with the findings by other authors for a-SiN_*x*_:H [30-32] and a-SiC_*x*_:H [33,34], where it has been attributed to a model based on band-tail states. In this model, the carriers radiatively recombine with the localized states at the band tails of the gap. With increasing nitrogen content, the bandgap energy increases, which results in a blue shift of the PL energy. However, for band-tail mechanism, the emission generally occurs at energies lower than 1.82 eV [11]. In this study, the peak position of PL spectra is higher than this value, especially for the samples with lower excess of Si. Furthermore, based on Robertson's calculated results [35,36], Ko et al. [37] have proposed possible mechanisms related to defect states to explain PL in silicon nitride films. Being amorphous, the samples have varying optical band gaps depending on composition. Therefore, the energy levels are not well defined. In addition, there is a distribution of states for a given defect giving rise to broad features in the PL spectra. Some investigations have shown both theoretically and experimentally that the PL band with peak positions of 1.8-3.2 eV [38-40] is closely related to the defect states within the bandgap of amorphous SiN_*x *_materials. Indeed, large amount of defect-related gap states generally exists in non-stoichiometric silicon nitride layers obtained by CVD process. These defect states having different energy levels contribute to the radiative emission by creating different channels for the relaxation of electronic states. It can be noticed that a weak peak appears around 3.1 eV, whose relative intensity decreases with increasing nitrogen content in the films. Wang et al. have attributed this peak to the presence of nitrogen-dangling bonds [41], whereas others have attributed it to the presence of ≡ Si^0 ^defect sites, which give gap states at 3.1 eV, 80% localized on sites [36]. In this case, as the band becomes prominent at higher Si content, it can be safely assigned to ≡Si^0 ^defect sites rather to than nitrogen-dangling bonds. From the above discussions, it can be ascertained that the predominant mechanism responsible for the PL behavior can be due to the recombination of defect states.

**Figure 5:**
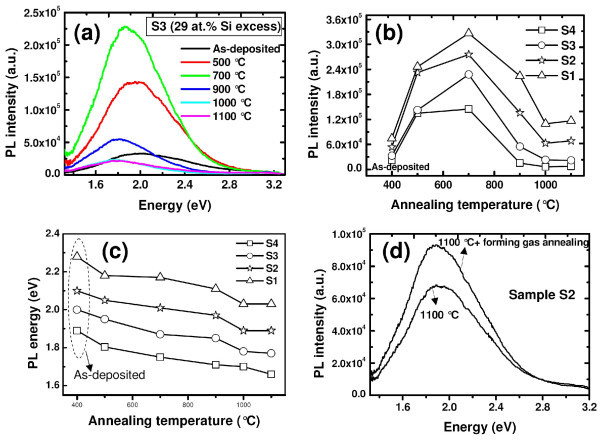
**Room temperature PL spectra**. **(a) **(Color online) Room temperature PL spectra of the sample S3 (29 at.% of silicon excess) subjected to thermal annealing within the temperature range of 500-1100°C, evolution of PL intensities **(b)**, and energies **(c) **as a function of annealing temperature, **(d) **evolution of PL intensity of the sample S2, which has been subjected to annealing at 1100°C in N_2_, and subsequently in forming gas (10% H_2 _+ 90% N_2_

Figure 5a shows the evolution of PL of sample S3 containing 29 at.% of excess Si with thermal annealing treatment of up to 1100°C. This evolution is similar for all other samples and is depicted in Figure 5b,c. As evident from Figure 5b, PL intensity increases and reaches a maximum at an annealing temperature of 700°C. A further increase in annealing temperature leads to a significant reduction of the PL intensity. The PL intensity drops to approximately 24% and approximately 10% of its peak value at 900 and 1100°C, respectively. The decrease in PL peak can be attributed to introduction of considerable amount of non-radiative defects after high-temperature annealing. It is reseaonable to expect more disordered structure, which is caused by the breaking of hydrogen bonds and subsequent effusion of hydrogen during high temperature thermal treatment. This phenomenon induces an increase in the number of dangling bonds, giving rise to an enhanced number of non-radiative recombination centers. Thus, the PL observed in the as-deposited sample quenches after high-temperature annealing above 700°C. In addition, a phase separation generally occurs when silicon-rich silicon nitride samples are annealed at high temperature (≥ 950°C), resulting in the formation of Si-nps embedded in a nearly stoichiometric silicon nitride matrix. Although, Si_3_N_4 _matrix itself could passivate some dangling bonds, causing non-radiative quenching, there are still a large number of dangling bonds existing in the film, especially at the interface region between Si-nps and Si_3_N_4 _matrix. Previously, it has been shown that one dangling bond is sufficient to quench the luminescence of a Si-np [42]. In this respect, the passivation of silicon- and nitrogen-dangling bonds acting as non-radiative recombination centers is an essential requirement for increasing the radiative yield without affecting the emission mechanism. In this regard, some previously annealed samples are subjected to an additional rapid thermal annealing in forming gas at 900°C for 1 min. Figure 5d shows the PL spectra of the sample S2, which has been subjected to annealing at 1100°C in N_2_, and subsequently in forming gas (10% H_2 _+ 90% N_2_). The PL peak intensity increases about 28% due to this additional forming gas annealing step, indicating passivation of some non-radiative recombination centers. It is noteworthy that the overall behavior of the PL band remains unchanged, indicating no change in the emission mechanism. Optimization of this hydrogen passivation process is currently under investigation.

### *C*-*V *and *G*-*V *measurement results

**Figure 6:**
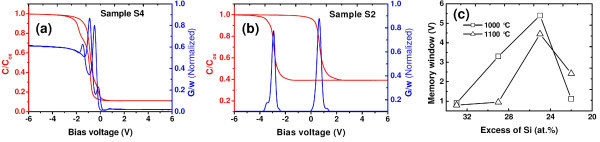
**(Color online) High-frequency (100 kHz) C-V characteristics of Al/Si_3_N_4_/SRSN/SiO_2_/Si (MNNOS) memory capacitors **of **(a) **the sample S4 containing 33 at.% of excess silicon, **(b) **the sample S2 containing 25 at.% of excess silicon, **(c) **evolution of memory window (calculated from flat-band shifts) versus excess silicon at two different annealing temperatures (1000 and 1100°C).

Figure 6a,b shows the typical high-frequency (100 kHz) *C*-*V *curves of Al/Si_3_N_4_/SRSN/SiO_2_/Si (MNNOS) capacitors with different excess amount of Si (33 and 25 at.%), and subjected to a post-thermal annealing at 1100°C in N_2 _ambient. All the capacitors show well-defined accumulation, depletion, and inversion regions in the *C*-*V *curves. Except S4 (33 at.% of excess Si), all other capacitors exhibit a sharp transition from accumulation and depletion to inversion, indicating the presence of less number of interface traps in the samples. In contrast, small irregularities or smear-out effect have been observed in the depletion region capacitance of sample S4, indicating the presence of some border (near-interfacial) traps in the MNNOS capacitor. All the capacitors show clockwise hysteresis, indicating a net positive charge (hole) trapping in the MNNOS capacitors [43]. The clockwise nature of *C*-*V *curves is generally attributed to charge storage through gate-injection mechanism. In fact, owing to rather thicker SiO_2 _interfacial layer, the substrate injection of carriers has been suppressed, which otherwise gives counterclockwise hysteresis loop. Under the influence of a positive bias voltage, holes are injected into the SRSN film from the top Al gate electrode, creating an abundance of holes in the SRSN charge-trapping layer. This promotes the injection of holes from the SRSN layer to the citrated Si-nps and/or defects inside the SRSN matrix, leading to a net hole trapping. Upon applying a negative bias voltage, the holes are subsequently flushed out (equivalent to injection of electrons) from the SRSN charge-trapping layer to the gate electrode, resulting in a negative flatband voltage shift and clockwise hysteresis loop. In addition, the presence of mobile ions and/or dielectric polarization can always give rise to a clockwise hysteresis. However, such a large amount of hysteresis window obtained at room temperature cannot be attributed to mobile charges, as their contribution is negligible at room temperature. In addition, the presence of mobile ions in the dielectric films leads to clockwise hysteresis, but no flatband shift in the positive sweep direction. This is inconsistent with the above results of *C*-*V *hysteresis, where a huge positive shift of flatband voltage has been noticed. Thus, the observed memory window can be safely assigned to charge storage in Si-nps and/or deep traps inside SRSN matrix. Contrary to what was expected, the sample S4 with the highest content of Si excess exhibits the lowest hysteretic effect, even though EFTEM images clearly indicate the existence of Si-nps. It is speculated that the reduced memory window is due to lateral charge loss through leakage paths introduced by insufficiently localized Si-nps and defect sites. It has been observed that the sample S2 with 25 at.% of Si excess exhibits the highest hysteretic effect, even though no Si-np was detected in our EFTEM analysis. An estimated memory window of 4.45 V was obtained at a sweep voltage of ± 8 V. The memory effect in the absence of Si-nps can be attributed to charge storage in deep level traps in the SRSN matrix and/or excess of Si at SRSN/SiO_2 _interface. Such type of charge storage has been obtained for SONOS-type memory structure having a SRSN charge-trapping layer [44,45]. Different models of electron and hole traps have been proposed for silicon nitride films. First, the model of dangling Si bonds as a capturing center [46,47]. Second, the presence of three fold coordinated negatively (K^- ^center) and positively (K^+ ^center) charged silicon atoms as traps for holes and electrons, respectively [48], and third to Si-Si bond or nitrogen vacancy possibly being responsible for electron and hole localization [49]. According to Robertson and Powell [36], SRSN films subjected to high-temperature annealing contain a significant amount of ≡ Si° defects, which behave as memory trap because it is amphoteric, deep, and energetically aligned with the gap of Si. These traps can trap either electrons or holes during programming, and release them during erase process. However, the trapping phenomena in silicon nitride are rather complicated and detailed investigation is in progress. As evident from Figure 6a,b, the conductance peak position in the samples are situated close to the flat-band voltage of the *C*-*V *curves. The separation of the conductance peak position during bidirectional sweep agrees well with the memory window values obtained from *C*-*V *measurements. Except the sample S4, all other samples exhibit single conductance peak in both forward and reverse sweep directions, indicating single electron charging effect. However, the sample S4 exhibits two peaks in bidirectional sweep, indicating sequential trapping and detrapping of two electrons [50]. The difference in peak heights during forward and reverse sweep can be attributed to different charge states of the traps in both sweep directions. Thus, we can conclude that the charge storage properties of SRSN layers can be enhanced by suitably tuning the Si excess content and annealing conditions. Figure 6c exhibits the variation of memory window for all the samples at two different annealing temperatures of 1000 and 1100°C. In our study, 1000°C was found to be the optimum annealing temperature for getting larger charge storage capacity in the MNNOS memory capacitors.

**Figure 7:**
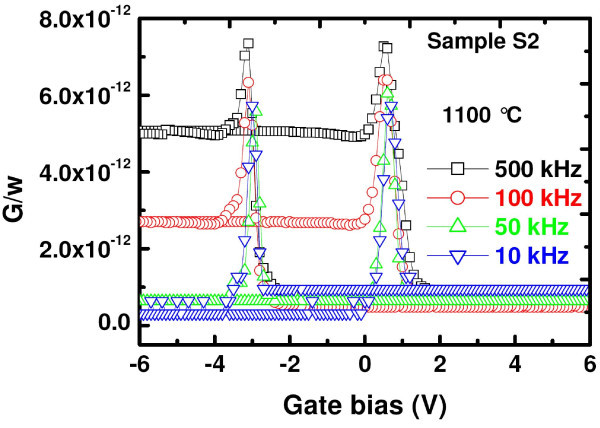
**(Color online) Frequency-dependent *G*-*V *characteristics of Al/Si_3_N_4_/SRSN/SiO_2_/Si (MNNOS) structure containing 25 at.% of Si excess in the range 10-500 kHz**.

For a better understanding of the results obtained from *C*-*V *measurements, frequency-dependent *G*-*V *measurements were further carried out in the frequency range of 10-500 kHz. Frequency-dependent *G*-*V *curves for the sample S2 are shown in Figure 7. *G*-*V *measurement is considered to be a more sensitive approach than *C*-*V *measurement technique, and provides the dynamic information related to trap density. In fact, conductance is related directly to the energy loss in response to the applied ac signal during the capture and emission of charge carriers by interface states. This method is quite effective even at high frequencies. The *G*-*V *curves exhibit a small parallel shift of 0.2 V along the voltage axis on decreasing the frequency from 500 to 10 kHz. This negligible shift of *G*-*V *curves can be due to the presence of a small quantity of fast traps in the memory capacitor. However, our frequency-dependent *C*-*V *curves remain almost constant with a change in measurement frequency (not shown here). Moreover, no distortion in *C*-*V *characteristics due to slow traps and/or large surface density (flat step) was observed in the samples with a change in frequency. It was noticed that the full width at half maxima (FWHM) of the conductance peak is small and almost constant in the frequency range of 10-500 kHz, indicating that the hysteresis and conductance peak are of the same origin. Furthermore, the conductance peak, where energy loss is maximum, increases with measurement frequency as more interface states at higher frequency could not respond to the change in ac signal resulting in a greater loss. From the above discussions, it can be ascertained that the charge storage is predominantly in deep traps inside the SRSN matrix and at SiO_2_/SRSN interface.

## Conclusions

In summary, a wide range of a-SiN_*x*_:H films with an excess of silicon (20 to 50%) with MW-PECVD system under the flows of NH_3 _and SiH_4 _have successfully been deposited. In addition, the silicon-rich a-SiN_*x*_:H films were sandwiched between a bottom thermal SiO_2 _and a top Si_3_N_4 _layer, and subsequently annealed within the temperature range of 500-1100°C in N_2 _ambient. A strong visible PL at room temperature has been observed for all the stack structures. The PL in the stack layers are predominantly due to defect states within the bandgap of silicon nitride. The highest PL intensity was obtained at an annealing temperature of 700°C. The decrease in PL intensity after 700°C is due to breaking of hydrogen bonds and effusion of hydrogen, which creates non-radiative centers. Amorphous Si quantum dots with an average size of about 3 to 3.6 nm were formed for the silicon nitride layer containing 33 at.% of silicon excess at an annealing temperature of 1100°C. The SRSN layer containing 25 at.% of excess silicon exhibits the highest memory window of 4. 45 V at a sweep voltage of ± 8 V at an optimum annealing temperature of 1000°C. Frequency-dependent *C*-*V *and *G*-*V *curves show that the charge storage is due to deep traps and minimal contribution from interface traps.

## Abbreviations

NH_3_: ammonia; *C*-*V*: capacitance-voltage; *G*-*V*: conductance-voltage; ERDA: elastic recoil detection analysis; ECR-PECVD: electron-cyclotron resonance plasma enhanced chemical vapor deposition; EFTEM: energy-filtered transmission electron microscopy; FTIR, Fourier transform infrared; FWHM: full width at half maxima; HREM, high resolution transmission electron microscopy; MNNOS: metal-nitride-nitride-silicon; MNOS, metal-nitride-oxide-silicon; MONOS: metal-oxide-nitride-oxide-silicon; NVMs: non-volatile charge memories; PL, photoluminescence; QCE: quantum confinement effect; RBS: Rutherford backscattering; SiH_4_: silane; Si-nps: silicon nanoparticles; SNOS: silicon-nitride-oxide-silicon; SONOS: silicon-oxide-nitride-oxide-silicon; SRSN: silicon-rich a-SiN_*x*_:H films; SRSN: silicon-rich silicon nitride.

## Competing interests

The authors declare that they have no competing interests.

## Authors' contributions

BSS and AS designed the study. BSS deposited the samples with the help of FD and GF. BSS investigated and performed all the post-fabrication treatment, carried out all the electrical characterization, FTIR studies, analyzed all the results, and prepared the draft of the manuscript. DM analyzed the RBS and ERDA measurement results. FD and BSS carried out the PL study. MC and FD carried out the TEM studies and investigated the results. Moreover, AS and BSS participated in the coordination of study. All authors read and approved the final manuscript.
